# Impact of Microbial Uptake on the Nutrient Plume around Marine Organic Particles: High-Resolution Numerical Analysis

**DOI:** 10.3390/microorganisms10102020

**Published:** 2022-10-13

**Authors:** George E. Kapellos, Hermann J. Eberl, Nicolas Kalogerakis, Patrick S. Doyle, Christakis A. Paraskeva

**Affiliations:** 1 Department of Chemical Engineering, Massachusetts Institute of Technology, Cambridge, MA 02139, USA; 2 Department of Chemical Engineering, University of Patras, 26504 Patras, Greece; 3 Department of Mathematics and Statistics, University of Guelph, Guelph, ON N1G 2W1, Canada; 4 School of Chemical and Environmental Engineering, Technical University of Crete, 73100 Chania, Greece

**Keywords:** ocean carbon cycle, nutrient uptake, marine snow aggregates, biological carbon pump, biodegradation, mass transfer, mathematical modeling

## Abstract

The interactions between marine bacteria and particulate matter play a pivotal role in the biogeochemical cycles of carbon and associated inorganic elements in the oceans. Eutrophic plumes typically form around nutrient-releasing particles and host intense bacterial activities. However, the potential of bacteria to reshape the nutrient plumes remains largely unexplored. We present a high-resolution numerical analysis for the impacts of nutrient uptake by free-living bacteria on the pattern of dissolution around slow-moving particles. At the single-particle level, the nutrient field is parameterized by the Péclet and Damköhler numbers (*0 < Pe < 1000*, *0 < Da < 10*) that quantify the relative contribution of advection, diffusion and uptake to nutrient transport. In spite of reducing the extent of the nutrient plume in the wake of the particle, bacterial uptake enhances the rates of particle dissolution and nutrient depletion. These effects are amplified when the uptake timescale is shorter than the plume lifetime (*Pe/Da < 100*, *Da > 0.0001*), while otherwise they are suppressed by advection or diffusion. Our analysis suggests that the quenching of eutrophic plumes is significant for individual phytoplankton cells, as well as marine aggregates with sizes ranging from 0.1 mm to 10 mm and sinking velocities up to 40 m per day. This microscale process has a large potential impact on microbial growth dynamics and nutrient cycling in marine ecosystems.

## 1. Introduction

The oceanic carbon cycle is modulated by the interactions between marine microbes and particulate organic matter (POM) via a multistep process known as the biological carbon pump [1,2,3]. In the euphotic zone, phytoplankton converts atmospheric CO_2_ to organic polymers that coagulate and form composite hydrogel particles called marine snow [4,5,6]. The polymeric matrix of marine snow hosts diverse microbial populations, embeds mineral particles, and concentrates metabolites, lysate and essential inorganic nutrients (N, P, Fe, Si) [6,7,8]. Being heavier than seawater, marine snow sinks and carries carbon to the seabed [9,10]. On the way, heterotrophic bacteria consume and mineralize a significant part, up to 60–80%, of the sinking marine snow back to CO_2_ [11]. Any change in the rates of these interlinked processes may disrupt the ecosystem’s balance. Global warming and elevated seawater temperature, for instance, may enhance the uptake of marine snow, increase the microbial abundance and, in turn, upregulate all levels of the marine food webs. However, this may also trigger a cascade of adverse effects, such as the reduction of carbon storage to the seabed, the expansion of oxygen minimum zones, and the acidification of seawater [12,13,14,15,16]. Improved mechanistic understanding of the bacteria-POM interactions across multiple spatial and temporal scales is key to a more sustainable management of our marine ecosystems.

**Figure 1:**
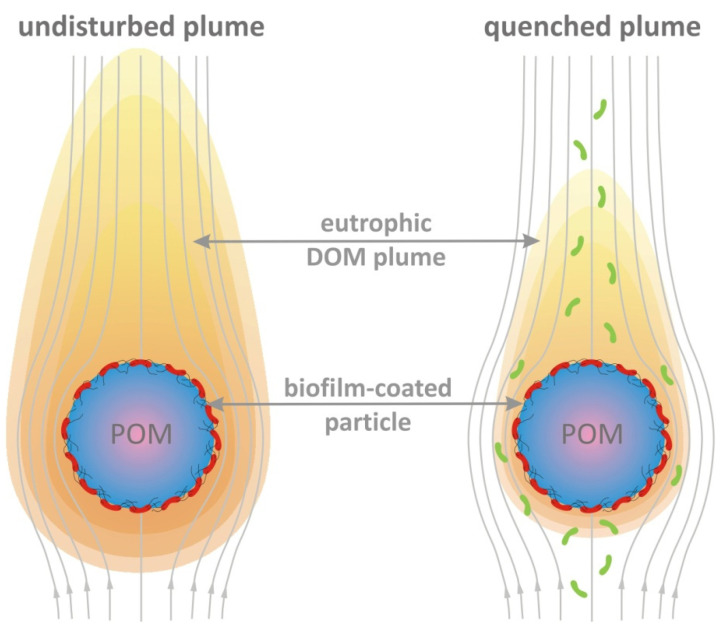
Conceptual illustration of nutrient plume quenching. The sinking particle (POM) creates a comet-shaped eutrophic plume of dissolved organic matter (DOM) with products from the enzymatic hydrolysis of particulate ingredients and the metabolic activities of surface-attached bacteria (red). Free-living bacteria (green) may act as point sinks, harvest useful solutes and reshape the nutrient field around the particle. The reduction in the extent and intensity of the plume is referred to as *plume quenching* and affects microbial foraging processes in the oceans. The plume length is critical in the detection of the organic particle by zooplankton [35] and the volume of the plume supports elevated growth rates for bacterioplankton [36].

Marine snow, phytoplankton cells and fecal pellets are the main indigenous sources of POM. Secondary exogenous sources are atmospheric and terrestrial depositions [17], as well as freshwater and estuarine particulates transferred by rivers into the sea [6,18]. Of emerging importance are oil droplets released by natural seeps or accidental spills [19,20,21] and microplastics stemming from anthropogenic pollution [22]. All such particles may serve as hotspots for the activities of both surface-attached [21,23] and free-living bacteria [8]. Biofilm-forming bacteria colonize the particle surface and affect the rates of POM degradation and solubilization in multiple ways (Figure 1). First, the extracellular polymeric matrix of the biofilm acts as a diffusive barrier and regulates the transport of solutes from the particle surface to ambient water, and vice versa [24]. Second, biofilm formers use exoenzymes and biosurfactants to transform refractory POM compounds into dissolved organic matter, part of which is taken up in the biofilm and the rest leaks out in ambient water [21,25,26,27,28,29,30,31]. Finally, the intense and highly diverse activity, typically established within natural multispecies biofilms, creates a rich stream of metabolic products that is also discharged into ambient water [32,33,34].

Solubilized POM and metabolites of the biofilm formers spread by diffusion and advection to form a nutrient-rich plume around the particle. The volume of the plume core may be 10–100 times the particle volume [37], with nutrient concentrations from one to three orders of magnitude higher than ambient levels (Table 5 in [6], [26,27]), thereby offering a unique nutritional opportunity to planktonic microbes [8,38,39,40]. Theoretical and experimental studies suggest that the utilization of nutrient plumes by free-living bacteria can substantially contribute to the global dynamics of microbial growth and carbon cycling in the oceans [36,40]. However, most analyses rely on the undisturbed nutrient field (i.e., zero-consumption) and the potential of free-living bacteria to reshape plumes has been overlooked. In principle, the contribution of bacterial uptake in shaping chemical fields is expected to be maximal in low flow microenvironments [41,42,43,44] and vanishing under strong turbulent mixing [45,46,47]. For sinking particles under moderate flow conditions, the extent of plume reshaping remains questionable [48]. To address this knowledge gap, we investigated numerically the impact of nutrient uptake by free-living bacteria on the dissolution pattern around slow-moving particles in a water column.

## 2. Materials and Methods

### 2.1. Nutrient Transport and Consumption at the Particle Scale

We consider a biofilm-coated organic particle as a rigid sphere that moves with constant velocity through an unbounded fluid domain (Figure 1). The moving particle generates a eutrophic trailing plume, which is quenched by free-living bacteria. In the particle frame of reference, the steady-state pattern of dissolution around the particle is described by the advection-diffusion equation coupled to non-homogeneous nutrient uptake, as expressed in the following dimensionless form:

(1) $${\mathrm{Pe}}_{A\upsilon }v_{\upsilon }\cdot \nabla C_{A\upsilon }=\nabla^{2}C_{A\upsilon }-{\mathrm{Da}}_{A\upsilon }f_{sat}C_{A\upsilon }$$

For the non-dimensionalization, the particle radius ${\tilde{R}}_{P}$ is the reference length, the average concentration ${\tilde{C}}_{ref}$ at the particle surface is the reference concentration, and the ambient water velocity ${\;\tilde{v}}_{\infty }$ is the reference velocity. The tilde (~) over a symbol denotes a dimensional quantity, whereas its absence denotes a dimensionless one. In Equation (1), $C_{A\upsilon }$ is the nutrient concentration, $v_{\upsilon }$ is the fluid velocity, $f_{sat}$ is the uptake saturation factor, ${\mathrm{Pe}}_{A\upsilon }={\;\tilde{v}}_{\infty }{\tilde{R}}_{P}/{\tilde{D}}_{A\upsilon }$ is the radius-based Péclet number, ${\mathrm{Da}}_{A\upsilon }={\tilde{k}}_{\infty }{\tilde{R}}_{P}^{2}/{\tilde{D}}_{A\upsilon }$ is the Damköhler number, ${\tilde{k}}_{\infty }$ is the uptake coefficient, and ${\tilde{D}}_{A\upsilon }$ is the nutrient diffusivity.

The uptake saturation factor is defined as $f_{sat}=1$ for linear unsaturated uptake, $f_{sat}=u_{max}/\left(K_{S}+C_{A\upsilon }\right)$ for Michaelis-Menten kinetics, and $f_{sat}=\min\left\{C_{sat}/C_{A\upsilon },1\right\}$ for Blackman’s bilinear kinetics [49], where $C_{sat}$ is the saturation threshold and $u_{max}$, $K_{S}$ are microbial kinetic parameters. For the presented Results, we used Blackman’s kinetics because it is simpler, monoparametric, and maintains consistency with previous relevant works, as all have considered linear kinetics [41,42,43,44,45,46,47].

### 2.2. Boundary Conditions on the Nutrient Field

Far from the particle, the nutrient concentration obtains a constant ambient value:

(2) $$C_{A\upsilon }\left(\left|x\right|\to \infty \right)=C_{A\infty }$$

At the particle surface, two alternative boundary conditions are considered [37]. For transport-limited dissolution, typically associated with partition equilibrium between the particulate and aqueous phases (e.g., oil-water), the solute concentration is prescribed over the particle surface:

(3) $$C_{A\upsilon }\left(\left|x\right|=1\right)=C_{As}$$

For reaction-limited dissolution, as for example associated with active exudation of metabolites by phytoplankton cells, the solute flux is prescribed over the particle surface:

(4) $$-{\frac{\partial C_{A\upsilon }}{\partial r}|}_{\left|x\right|=1}=q_{As}$$

where $q_{As}={\tilde{q}}_{As}/{\tilde{q}}_{ref}$ and ${\tilde{q}}_{ref}={\tilde{C}}_{ref}{\tilde{D}}_{A\upsilon }/{\tilde{R}}_{P}$ is the reference flux.

By comparison, the concentration is constant and the flux varies with the Péclet and Damköhler numbers in transport-limited dissolution, whereas the flux is constant and the concentration varies over the particle surface in reaction-limited dissolution. Results in Section 3.4 were obtained for transport-limited dissolution with partition equilibrium at the particle-water interface ($C_{As}=1$), and complementary results in Section 3.5 were obtained for reaction-limited dissolution. Far from the particle, the ambient nutrient concentration is considered much smaller than the concentration on the particle surface ($C_{A\infty }=0$). In eutrophic coastal and upwelling waters, even when the average nutrient concentration in ambient water is appreciable on the ocean sampling scale, microscale advection and elevated consumption sustain sharp nutrient gradients at the particle scale and hence it is reasonable to consider that $C_{A\infty }\ll C_{As}$ in most cases.

### 2.3. Flow Field around the Particle

For slow-moving microparticles, the flow around the particle is laminar with low Reynolds number, Re=ρ˜υ v˜∞R˜P/μ˜υ<1, where ${\tilde{\rho }}_{\upsilon }$ is the density of ambient water, ${\tilde{\mu }}_{\upsilon }$ is the dynamic viscosity of ambient water, and ${\;\tilde{v}}_{\infty }$ is the average velocity of ambient water relative to the particle. Thus, the radial and angular components of the dimensionless velocity vector, $v_{\upsilon }$, are given by Stokes’ solution [24]:

(5a) $$v_{\upsilon ,r}(r,\theta )=-\left(1-\frac{3}{2r}+\frac{1}{2r^{3}}\right)\cos\theta$$

(5b) $$v_{\upsilon ,\theta }(r,\theta )=\left(1-\frac{3}{4r}-\frac{1}{4r^{3}}\right)\sin\theta$$

### 2.4. Plume Metrics

The ratio of the plume volume over the particle volume is defined as

(6) $$V_{plm}\equiv \frac{{\tilde{V}}_{plm}}{{\tilde{V}}_{P}}=\frac{3}{4\pi }\int_{V_{\upsilon }}^{}H(\bar{c})dV$$

where $H\left(\bar{c}\right)$ is the Heaviside function, with $H\left(\bar{c}\right)=1$ if $\bar{c}>0$ and nil otherwise, $\bar{c}=C_{A\upsilon }-C_{det}$ and $C_{det}$ is the threshold concentration that defines the detectable plume. The integration in Equation (6) is carried out over the entire volume of ambient fluid with the dimensionless differential volume $dV=r^{2}\sin\theta drd\theta d\varphi$ in spherical coordinates. The plume length is defined as the distance from the particle center at which the nutrient concentration in the wake of the particle (θ=π) is equal to the detection threshold:

(7) $$C_{A\upsilon }\left(L_{plm},\pi \right)=C_{det}$$

Analytical correlations, which are useful for engineering applications, have been established for the plume length and volume by representing the nutrient-releasing particle as a point source [35,48]:

(8) $$L_{plm}^{*}=\frac{{\mathrm{Sh}}_{R}}{C_{det}}\exp\left(-\frac{\mathrm{Da}}{\mathrm{Pe}}L_{plm}^{*}\right)$$

(9) $$V_{plm}^{*}=\frac{3L_{plm}^{*2}}{4\mathrm{Pe}}\left(1+\frac{2}{3}\frac{\mathrm{Da}}{\mathrm{Pe}}L_{plm}^{*}\right)$$

For Da>0, Equation (8) is nonlinear and can be solved either graphically or by using a root finding method. Compared with our numerical results, Equation (9) underestimates the plume volume by less than 30% for Pe>max{1,Da}.

### 2.5. Efficiencies of Dissolution and Degradation

The total nutrient flux through a spherical surface with radius *r* from the center of the particle is defined as:

(10) $$Q_{As}(r)\equiv \frac{{\tilde{Q}}_{As}(\tilde{r})}{{\tilde{q}}_{ref}{\tilde{R}}_{P}^{2}}=\int_{S}^{}q_{A}\cdot e_{r}dS$$

where ${\tilde{q}}_{ref}={\tilde{C}}_{ref}{\tilde{D}}_{A\upsilon }/{\tilde{R}}_{P}$ is the reference flux, $q_{A}={\mathrm{Pe}}_{A\upsilon }v_{\upsilon }C_{A\upsilon }-\nabla C_{A\upsilon }$ is the combined nutrient flux that accounts for both advection and diffusion, and $dS=r^{2}\sin\theta d\theta d\varphi$ is the differential area on the spherical surface. In the engineering literature, the overall dissolution rate at the particle surface is usually expressed as ${\tilde{Q}}_{As}={\tilde{k}}_{As}{\tilde{S}}_{P}{\tilde{C}}_{ref}$, where ${\tilde{S}}_{P}=4\pi {\tilde{R}}_{P}^{2}$ is the particle surface area and ${\tilde{k}}_{As}=\left({\tilde{D}}_{A\upsilon }/{\tilde{R}}_{P}\right)Q_{As}/4\pi$ is the mass transfer coefficient. In turn, the radius-based Sherwood number is defined as:

(11) $${\mathrm{Sh}}_{R}\equiv \frac{{\tilde{k}}_{As}{\tilde{R}}_{P}}{{\tilde{D}}_{A\upsilon }}=\frac{Q_{As}(1)}{4\pi }$$

and represents the dimensionless speed of dissolution. The Sherwood number may equivalently be expressed as ${\mathrm{Sh}}_{R}={\tilde{Q}}_{As}/\left(4\pi {\tilde{R}}_{P}{\tilde{D}}_{A\upsilon }{\tilde{C}}_{ref}\right)$ and represent the ratio of the total nutrient flux, inclusive of advection and consumption effects, over the diffusive nutrient flux alone. The dissolution enhancement that is caused by nutrient consumption is calculated as:

(12) $$E_{dis}=\frac{{\mathrm{Sh}}_{R}}{{\mathrm{Sh}}_{R0}}$$

where ${\mathrm{Sh}}_{R0}$ is the Sherwood number in the absence of uptake (Da=0). The degradation efficiency is the fraction of released nutrient that is consumed within a spherical shell of outer radius *r*:

(13) $$E_{deg}(r)=1-\frac{Q_{As}(r)}{Q_{As}(1)}$$

### 2.6. Description of the High-Resolution Numerical Scheme

The advection-diffusion-bioreaction equation given in Equation (1) is solved numerically with a finite difference method in spherical coordinates. As there is no driving force for a change in the concentration along the azimuthal direction, the solution is axisymmetric and suffices to discretize the partial differential equation on the (r, θ) plane. The first and second-order partial derivatives in the diffusion operator are discretized with the central difference scheme of *Sundqvist & Veronis* [50]. The first-order derivatives in the advection operator are discretized with the third-order upwind scheme of *Liu* et al. [51]. This combination successfully suppresses numerical diffusion at low Péclet numbers and non-physical oscillations at high Péclet numbers. To further enhance the stability of the scheme, the Dirichlet boundary condition given in Equation (2) is replaced by a non-reflecting Neumann condition, $dC_{A\upsilon }/dr=0$, on the outflow part ($v_{\upsilon ,r}>0$) of the outer boundary for Pe ≥ 0.1. Finite differences transform Equation (1) into a sparse system of nonlinear algebraic equations, which is solved with the Picard iterative method. At each iteration, a linearized system is solved with the BiCGstab method [52].

The computational domain is discretized with a body-conforming non-uniform grid, which consists of concentric circles and perpendicular rays. In the polar direction, the grid spacing $d_{\theta }$ is constant in the upstream region ($0<\theta <\theta_{g}$) and decreases geometrically in the downstream region ($\theta_{g}<\theta <\pi$). In the radial *r*-direction, the grid spacing $d_{r}$ increases geometrically for 1 < *r* < *R_g_* and then increases linearly up to the outer boundary (*R_g_* < *r* < *R_∞_*). This grid design provides high spatial resolution both around the particle and also far downstream, thereby capturing the fine features of the boundary layer on the particle surface and of the plume’s tail in the wake of the particle.

**Figure 2:**
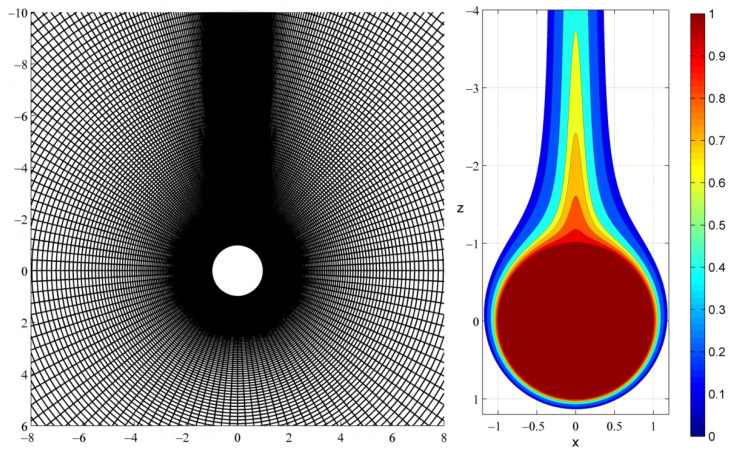
Representative computational grid and detail of the trailing plume for Pe = 1000. The computational grid is dense around and behind the particle so as to capture both the boundary layer and the plume tail with sufficiently high numerical resolution.

For the calculations presented in the Results, the grid parameters were optimized to achieve high accuracy at reasonable computational time (Figure 2). For Pe ≥ 0.1, the outer boundary was set at R_∞_ = 101 and the grid consisted of 621 × 253 computational elements along the radial and polar directions, respectively. The angle of transition in the polar spacing was $\theta_{g}=\pi /2$, and the radius of transition in the radial spacing was *R_g_* = 15. In the polar direction, the spacing was maximum $d_{\theta }=\pi /72$ at the upstream pole (θ=0) and minimum $d_{\theta }=10^{-4}$ at the downstream pole (θ=π). In the radial direction, the grid spacing was minimum $d_{r}=5\times 10^{-4}$ at the particle surface (*r* = 1) and maximum $d_{r}=0.375$ at the outer boundary (*r* = *R_∞_*). The thickness of the boundary layer scales with $Pe^{1/3}$. The grid contains 85 elements in a layer of thickness 0.1 (Pe = 1000) around the particle. For the resolution of the plume’s tip at a distance of *r* = 60 in the wake of the particle, the grid contains 60 elements within a chord of length 0.1 (Pe = 1000). For Pe < 0.1 and Pe > 1000, the outer boundary was set at *R_∞_* = 201. To ensure that the results are grid-independent, computations were also made with coarser and finer grids.

## 3. Results and Discussion

### 3.1. Validation of the Numerical Methodology

The accuracy of our numerical scheme was verified by comparing our calculations for the Sherwood number with available data from the literature. For zero consumption (Da = 0), excellent agreement is observed with published data and correlations. The correlation of *Clift* et al. [53] works perfectly over the entire range of Péclet:

(14) $${\mathrm{Sh}}_{R0}=\frac{1}{2}\left[1+{\left(1+2{\mathrm{Pe}}_{R}\right)}^{1/3}\right]$$

**Figure 3:**
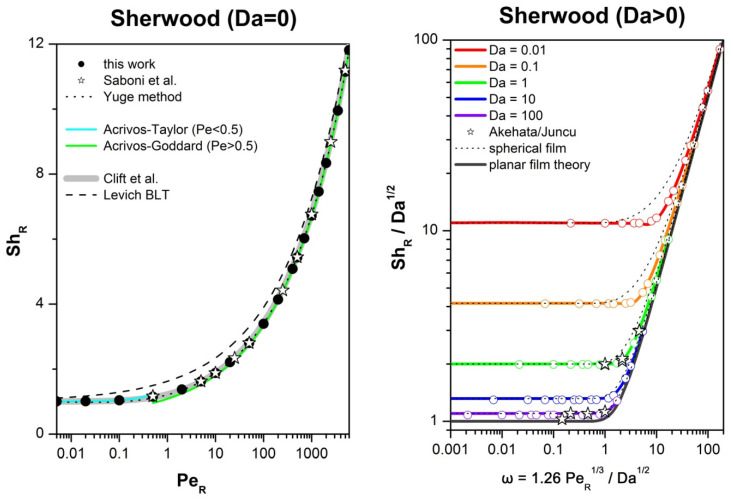
Validation of the numerical model against published data for the radius-based Sherwood number, as a function of the Péclet and Damköhler numbers within the range defined in Table 1. For zero consumption (Da = 0), our calculations are in excellent agreement with numerical data and correlations from the literature [53,56,57,58,59]. For finite consumption (Da > 0), our calculations (circles) are in good agreement with the Yuge approximation (colored lines), numerical data (stars) [54,60], the planar film theory (black line) and the spherical film theory (black dotted lines) [55]. In addition to the correlations given in the main text, we have: ${\mathrm{Sh}}_{R0}=1+0.6245Pe_{R}^{1/3}$ (dashed line [56]), ${\mathrm{Sh}}_{R0}=0.461+0.6245Pe_{R}^{1/3}$ for Pe > 0.5 (green line [57]), and ${\mathrm{Sh}}_{R0}=1+0.5Pe_{R}+0.5Pe_{R}^{2}\ln\left(Pe\right)$ for Pe < 0.5 (cyan line [58]).

Furthermore, for zero consumption, mass conservation demands that $E_{deg}=0$ for $1\leq r\leq R_{\infty }$. This constraint was also used to assess the overall performance and ability of our numerical scheme to preserve the mass balance. A maximum deviation of $\left|E_{deg}\right|<10^{-3}$ was achieved in all simulations. For finite consumption (Da > 0), our calculations are in excellent agreement with the Yuge approximation [54] and available numerical data (Figure 3). The planar film theory, ${\mathrm{Sh}}_{R}=\sqrt{\mathrm{Da}}/\tanh\left(2/\omega \right)$ with $\omega =1.26{\mathrm{Pe}}_{R}^{1/3}/\sqrt{\mathrm{Da}}$, is applicable only if ${\mathrm{Pe}}_{R}>100$ or Da>100, while the spherical film theory [55]:

(15) $${\mathrm{Sh}}_{R}=1+\sqrt{\mathrm{Da}}/\tanh(2/\omega )$$

is acceptable over the entire range of Péclet and Damköhler for engineering applications.

### 3.2. Fundamental Timescales and Dimensionless Numbers

The nutrient field around marine organic particles is shaped by the interplay between advection, diffusion and uptake by microorganisms. The relative importance of these processes is quantified by the dimensionless Péclet and Damköhler numbers. In particular, the Péclet number, $\mathrm{Pe}={\;\tilde{v}}_{\infty }{\tilde{R}}_{P}/{\tilde{D}}_{A\upsilon }$, is the ratio of advective over diffusive transport rates and captures the combined effect of the particle size, the particle velocity, and the nutrient diffusivity. Similarly, the Damköhler number, $\mathrm{Da}={\tilde{k}}_{\infty }{\tilde{R}}_{P}^{2}/{\tilde{D}}_{A\upsilon }$, is the ratio of the uptake rate over the diffusive transport rate. In terms of fundamental timescales, the Péclet and Damköhler numbers can be expressed as $\mathrm{Pe}={\tilde{\tau }}_{D}/{\tilde{\tau }}_{A}$ and $\mathrm{Da}={\tilde{\tau }}_{D}/{\tilde{\tau }}_{U}$, respectively, where ${\tilde{\tau }}_{D}={\tilde{R}}_{P}^{2}/{\tilde{D}}_{A\upsilon }$ is the diffusion timescale, ${\tilde{\tau }}_{A}={\tilde{R}}_{P}/{\;\tilde{v}}_{\infty }$ is the advection timescale, and ${\tilde{\tau }}_{U}={\tilde{k}}_{\infty }^{-1}$ is the uptake timescale. Table 1 lists the physically relevant range of values for the key system parameters engaged in timescale relations. The Péclet number is suitable for slow-moving microparticles, and the Damköhler number is on the high end for heterotrophic bacteria.

### 3.3. Nutrient Uptake Kinetics, Affinity and Timescale

The uptake timescale and, thus, the Damköhler number depend on the mechanisms of nutrient uptake. Bacteria acquire nutrients in a two-step process that involves the physical transport of solute molecules from the surroundings to the cell surface and, thereafter, the trans-membrane transport from the extracellular to the intracellular space. Physical transport is dominated by molecular diffusion, while advection is usually very weak at the bacterial length scale [62]. Trans-membrane transport relies on three main mechanisms: (1) passive diffusion through the lipid bilayer of small non-polar molecules, like oxygen and carbon dioxide; (2) facilitated diffusion through channel proteins (*porins*) of water and selected ions, like sodium and potassium; and (3) active intake by carrier proteins (*porters*) of large or polar molecules, like amino acids and sugars. Uptake of most organic nutrients is mediated by porters and the uptake rate per single-cell, ${\tilde{u}}_{A}$, is typically described by Michaelis−Menten kinetics ${\tilde{u}}_{A}={\tilde{u}}_{max}{\tilde{C}}_{A\upsilon }/\left({\tilde{K}}_{S}+{\tilde{C}}_{A\upsilon }\right)$, where ${\tilde{u}}_{max}$ is the maximum uptake rate and ${\tilde{K}}_{S}$ is the half-saturation constant. The kinetic parameters, ${\tilde{u}}_{max}$ and ${\tilde{K}}_{S}$, depend on microbial traits (i.e., cell size and shape, number of porters, handling time of nutrients by porters), as well as on the extracellular flow, the nutrient diffusivity, and the temperature [63,64,65].

At high nutrient levels, ${\tilde{C}}_{A\upsilon }\gg {\tilde{K}}_{S}$, the membrane transport system is saturated and the uptake rate obtains its maximum value ${\tilde{u}}_{A}={\tilde{u}}_{max}$. At low nutrient levels, ${\tilde{C}}_{A\upsilon }\ll {\tilde{K}}_{S}$, the uptake rate becomes ${\tilde{u}}_{A}={\tilde{\alpha }}_{S}{\tilde{C}}_{A\upsilon }$ where ${\tilde{\alpha }}_{S}={\tilde{u}}_{max}/{\tilde{K}}_{S}$ is the nutrient affinity, or clearance rate, and stands for the fluid volume that is swept of nutrients by porters per unit time. An upper bound for the nutrient affinity is ${\tilde{\alpha }}_{S,max}=4\pi {\tilde{R}}_{cell}{\tilde{D}}_{A\upsilon }$ [64] and has been established for spherical cells that act as perfect absorbers (i.e., efficiently uptake any nutrient molecules reaching their surface). For bacteria with an equivalent spherical radius of ${\tilde{R}}_{cell}=1\mu m$ and nutrients with diffusivity of ${\tilde{D}}_{A\upsilon }=10^{-5}cm^{2}/s$, the maximum affinity is ${\tilde{\alpha }}_{S,max}\sim 12pL/\left(cell\cdot s\right)$. Reported data for the bacterial affinity are tabulated below and range from tenths of femtoliters up to a few picoliters per second per cell. The experimental values are between 10% to 60% of the corresponding theoretical estimates, with acclimated bacteria showing better performance.

The uptake timescale is estimated by the inverse uptake coefficient, ${\tilde{\tau }}_{U}={\tilde{k}}_{\infty }^{-1}$, and relates to the nutrient affinity as ${\tilde{\tau }}_{U}={\left({\tilde{\alpha }}_{S}{\tilde{B}}_{\upsilon }^{\infty }\right)}^{-1}$, where ${\tilde{B}}_{\upsilon }^{\infty }$ is the average concentration of free-living bacteria in ambient water and ranges from 10^4^ cells/mL in the deep ocean to 10^7^ cells/mL in coastal waters [6,61]. In the presence of organic particles, the bacterial concentration is expected on the high end of pertinent data, ${\tilde{B}}_{\upsilon }^{\infty }\sim 10^{6}-10^{7}cells/mL$, because the elevated nutrient concentrations upregulate microbial growth rates [36] and the hydrodynamic interactions trap microbes in close proximity to the particles [66]. For particles with sizes in the range of ${\tilde{R}}_{P}=0.1-1mm$, small nutrient molecules with diffusivities on the order of ${\tilde{D}}_{A\upsilon }=10^{-5}cm^{2}/s$, and bacteria with affinities in the range of ${\tilde{\alpha }}_{S}=1-10pL/\left(cell\cdot s\right)$, both of the uptake and diffusion timescales are on the order of ${\tilde{\tau }}_{U}$, ${\tilde{\tau }}_{D}$ ~ $10-10^{3}s$, and the Damköhler number falls in the range of Da~0.01−100. The Damköhler number is generally invariant to the diffusion coefficient, because ${\tilde{\alpha }}_{S}\propto {\tilde{D}}_{A\upsilon }$ and any change in the diffusivity is cancelled out by a concomitant change in the uptake affinity (see Table 2). For example, large solutes with diffusivities on the the order of ${\tilde{D}}_{A\upsilon }=10^{-6}cm^{2}/s$ are uptaken by bacteria with lower affinities, ${\tilde{\alpha }}_{S}=0.1-1pL/\left(cell\cdot s\right)$, and the Damköhler range remains unchanged.

With regard to the data listed in Table 2, most sources provide the maximum uptake rate, ${\tilde{u}}_{max}$, per milligrams of wet cell weight. Where necessary, the following conversion factors were used: 0.3 (g dry cell weight) per (g wet cell weight), 5.7 (g wet cell weight) per (g protein) [79], 0.17 pg-carbon per µm^3^ [69], and 0.1 fmol-phosphorus per µm^3^ [67]. The per-biomass affinity was calculated as ${\tilde{\alpha }}_{Sb}={\tilde{u}}_{max}/{\tilde{K}}_{S}$ and the per-cell affinity as ${\tilde{\alpha }}_{S}={\tilde{\alpha }}_{Sb}{\tilde{m}}_{cell}$, where the single-cell wet mass, ${\tilde{m}}_{cell}$, was estimated from the cell density and volume upon assuming an average cell density of 1.1 g/mL [80].

### 3.4. Visualization and Metrics of Plume Quenching

**Figure 4:**
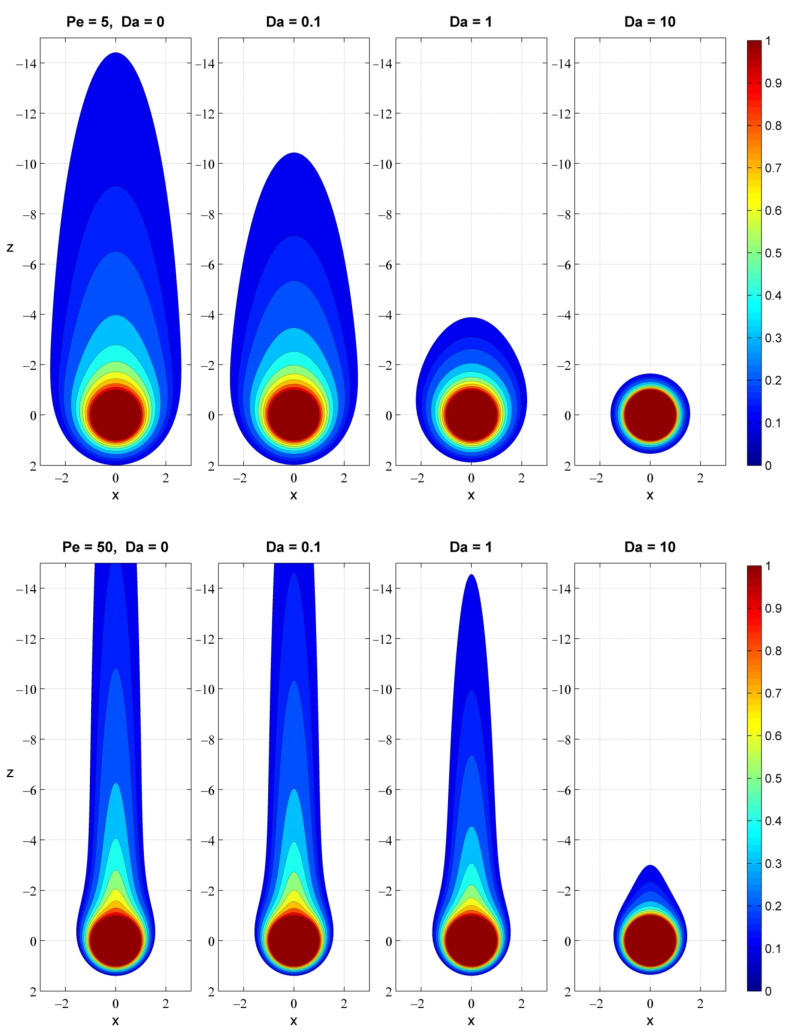
Effect of uptake (Da) on the nutrient concentration field around a slow-sinking particle with Pe = 5 (**top**) and Pe = 50 (**bottom**). The color represents nutrient level. Isoconcentration lines are shown at selected values of $C_{A\upsilon }$ (0.1, 0.15, and 0.2–1.0 with step 0.1). The z- and x-axes measure the dimensionless distance from the particle center.

**Figure 5:**
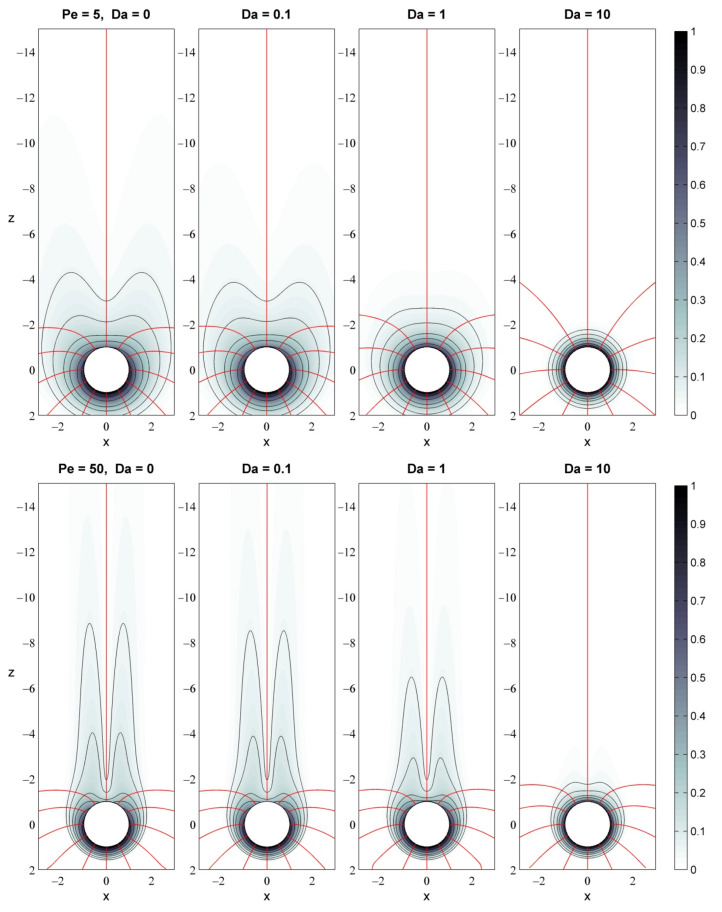
Effect of uptake (Da) on the diffusive flux field, $-\nabla C_{A\upsilon }$, around a slow-sinking particle with Pe = 5 (**top**) and Pe = 50 (**bottom**). The grayscale represents the magnitude of the flux vector, normalized by the maximum flux in each case. Black isoflux lines are shown at selected values (0.05, 0.1, 0.2, 0.3, 0.5, 0.7 and 0.9). Red fluxlines start at θ=kπ/6, with *k* = 0, …, 11 and specify the direction of the flux field. In analogy to fluid streamlines, nutrient fluxlines are tangent to the flux vector and define the paths followed by nutrient parcels leaving from the particle surface. The z- and x-axes measure the dimensionless distance from the particle center.

Given the physically relevant range of Péclet and Damköhler, we now examine the effects of advection (Pe) and uptake (Da) on the pattern and characteristic metrics of the nutrient field around slow-sinking particles. Figure 4 shows that isotropic diffusion, at a low Péclet number, spreads the nutrients into a spheroidal plume around the particle. Enhanced advection, at a higher Péclet number, entrains the nutrients into an elongated plume in the wake of the particle. The diffusive flux field, $-\nabla C_{A\upsilon }$, displays an intriguing bunny-face pattern (Figure 5), in which advection stretches out the bunny ears along the downstream direction. In all cases, nutrient uptake reduces the extent of the eutrophic trailing plume and smoothes out the delicate features of the concentration gradient, with increasing impact as the ratio of Pe/Da decreases. This combination of Damköhler with Péclet, $Pe/Da={\;\tilde{v}}_{\infty }{\tilde{\tau }}_{U}/{\tilde{R}}_{P}$, defines a new critical parameter that represents the ratio of the advective transport rate over the uptake rate and captures the combined effects of particle velocity, particle size and uptake timescale.

**Figure 6:**
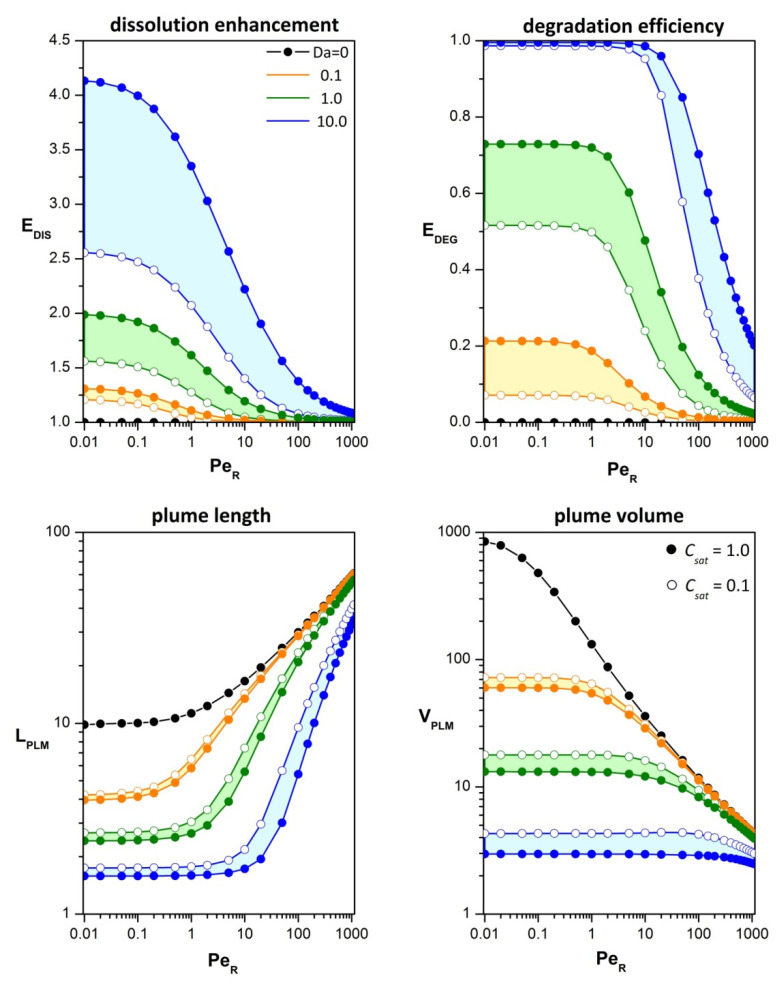
Effects of advection (Pe), uptake (Da) and saturation (*C_sat_*) on characteristic metrics of the nutrient field: dissolution enhancement (*E_dis_*), degradation efficiency at *r* = 3 (*E_deg_*), plume length (*L_plm_*), and plume volume (*V_plm_*). The detection threshold is *C_det_* = 0.1, and the saturation threshold is *C_sat_* = 1 for filled circles (unsaturable uptake) and *C_sat_* = 0.1 for open circles. The color shading highlights the saturation impact on each metric for three Damkohler numbers: Da = 0.1 (yellow), Da = 1 (green), and Da = 10 (cyan).

Figure 6 presents the effects of advection (Pe) and uptake (Da) on characteristic metrics of the nutrient field. Under partition equilibrium, both advection and uptake sharpen the nutrient distribution adjacent to the particle, especially at the upstream part, and increase the speed of dissolution. Consequently, fast uptake may cause a several-fold increase in the dissolution rate (*E_dis_*), but the effect is suppressed as advection gets stronger. A similar trend is observed for the degradation efficiency (*E_deg_*). At low Pe/Da ratios, elevated consumption in the vicinity of the particle (*E_deg_*~1) implies that the global degradation rate is limited by the dissolution rate. On the other hand, negligible degradation efficiency (*E_deg_*~0), at high Pe/Da ratios, means that the global degradation rate is limited by the uptake kinetics and nutrients dissipate into ambient water.

Compared to the plume metrics at low-Péclet numbers, strong advection may increase the plume length by an order of magnitude and decrease the plume volume by two orders. Concurrent nutrient uptake results in quenching of the plume extent, with significant impact for Pe/Da < 100. The phenomenon of saturation is also important and, as expected, mitigates the impacts of uptake. As shown in Figure 6, the saturation effect becomes more pronounced with increasing Damköhler number. The dependence of the plume metrics on saturation is nonlinear because the saturation threshold affects both the maximum uptake rate and the volume in which the rate is limited.

### 3.5. Regulation of Plume Formation by Particle-Associated Bacteria

**Figure 7:**
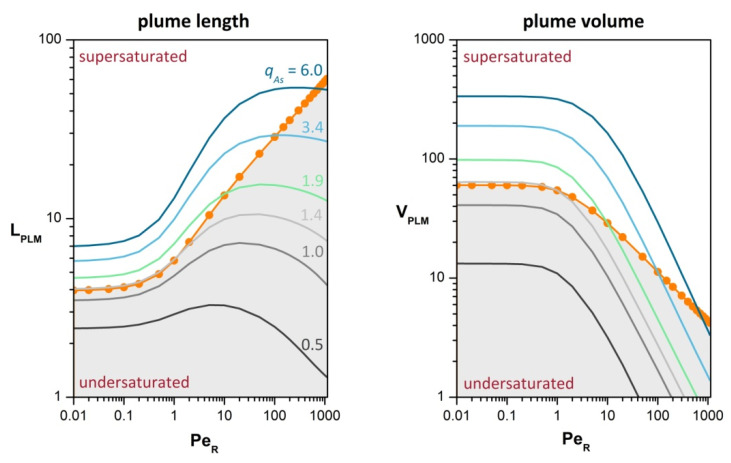
Effects of controlled release on the length ($L_{plm}$) and volume ($V_{plm}$ ) of the eutrophic plume around a nutrient-releasing particle. Higher release rate ($q_{As}$ ) represents elevated activities of particle-associated bacteria. The orange line with filled circles corresponds to transport-limited dissolution and represents the *uncoupled hydrolysis* concept set forth by Azam and coworkers [6,25,39], in which the enzymatic capacity significantly exceeds intraparticle uptake and leakage to ambient water. The orange line also delimits the domains of supersaturated ($C_{As}>1$) and undersaturated particles ($C_{As}<1$). Plume metrics were calculated for Da=0.1 and $C_{det}=0.1$ in all cases. The reference state ($q_{As}=1$ ) corresponds to a saturated particle in equilibrium with the surrounding water (Pe=0, Da=0 ).

For biofilm-coated particles, the enzymatic and metabolic activities of the surface- attached bacteria modulate the extent, intensity and composition of the eutrophic plume around the particle (Figure 7). Experimental studies with marine aggregates suggest that readily bioavailable nutrients, like amino and fatty acids, are enzymatically cleaved from the extracellular biopolymer matrix much faster than utilized by bacteria on the particle [6,25,26,27,28,29,39]. This phenomenon is known as uncoupled hydrolysis [6,25] and may result in particles fully saturated with solubilized nutrients. Under such conditions, partition equilibrium reasonably applies at the particle-water interface and plume formation is determined by transport in ambient water. Furthermore, in a recent experimental study, *Alcolombri* et al. [31] investigated the degradation of alginate microparticles by surface- attached bacteria and found that the release of solubilized oligo-alginate increases with increasing flow velocity (i.e., Péclet number). This trend is also captured by transport- limited dissolution, which inherently features a Péclet-dependent release rate (Figure 2). In this regard, the results presented in the previous section are most suitable for fresh aggregates enriched with bioavailable nutrients and high enzymatic capacity.

However, the microbiological and chemical composition of biofilm-coated particles changes with time, on the order of hours to days, due to microbial activities, succession dynamics, cell lysis, zooplankton grazing, absorption and desorption of cells, solutes and elementary particles from and to the surrounding water [6,7,8,23,38,81,82,83]. Intraparticle enzymatic activities also change [28,29]. For example, proteins and organic phosphorus are preferentially hydrolyzed in fresh marine aggregates, whereas polysaccharides are released in later stages by mature aggregates [28]. In conditions of limited enzymatic capacity, the nutrient release rate is tightly coupled to the intraparticle bacterial activities and basic insight into plume formation can be obtained through the boundary condition given in Equation (4) for reaction-limited dissolution. As shown in Figure 7, the interplay between nutrient release and advection may result in either supersaturated particles with large plumes or undersaturated particles with small plumes, as compared to the standard set by transport-limited dissolution. Interestingly, under controlled nutrient release, the eutrophic plume attenuates non-linearly with lowering release rate. For aggregates with marked spatial heterogeneity of bacterial activities, full resolution of the biochemical coupling between particle-associated and free-living bacteria would require to combine the present model formulation with models of intraparticle transport [24,44] and the concomitant biofilm dynamics [84,85].

### 3.6. Ecological Significance of Nutrient Plume Quenching

Plume quenching is plausible when the nutrient uptake rate is faster than the plume dissipation rate by advection and diffusion. In terms of fundamental timescales, the uptake timescale, ${\tilde{\tau }}_{U}$, must be shorter than the lifetime of the plume, ${\tilde{\tau }}_{plm}$. For advection dominated transport (Pe > 1), the plume lifetime is on the order of ${\tilde{\tau }}_{plm}={\tilde{L}}_{plm}/{\;\tilde{v}}_{\infty }$ [48] and, thus, the timescale condition ${\tilde{\tau }}_{U}<{\tilde{\tau }}_{plm}$ can be expressed in terms of the particle- based Péclet and Damköhler numbers as $Pe/Da<L_{plm}$. This is a nonlinear relation as the plume length also depends on the Péclet and Damköhler numbers. However, for the parameter range of interest, our numerical analysis has shown that it suffices to set Pe/Da<100 (Figure 6). Back-substitution of dimensional quantities, results in an upper bound for the applicable particle velocity, ${\;\tilde{v}}_{\infty }<\left(100/{\tilde{\tau }}_{U}\right){\tilde{R}}_{P}$, in relation to the particle size and the uptake timescale.

For diffusion-dominated transport (Pe < 1), the plume lifetime is on the order of ${\tilde{\tau }}_{plm}={\tilde{L}}_{plm}^{2}/{\tilde{D}}_{A\upsilon }$ and the timescale condition ${\tilde{\tau }}_{U}<{\tilde{\tau }}_{plm}$ can be expressed as $Da>L_{plm}^{-2}$. In practice, a measurable change (>5%) of the plume metrics is achieved when $Da>10^{-4}$ and sets a lower bound on the applicable particle size, ${\tilde{R}}_{P}>0.01\sqrt{{\tilde{\tau }}_{U}{\tilde{D}}_{A\upsilon }}$. For reasonably fast uptake (${\tilde{\tau }}_{U}=100s$) of small nutrient molecules (${\tilde{D}}_{A\upsilon }=10^{-5}cm^{2}/s$), plume quenching is expected to be substantial when the particle radius is over 3 μm. For larger nutrient molecules (${\tilde{D}}_{A\upsilon }=10^{-6}cm^{2}/s$), the radius limit falls to 1 μm. Therefore, the competition between uptake and diffusion is of relevance to individual phytoplankton cells [62,86].

Another constraint is set upon the applicable particle types by the low Reynolds condition, $Re={\tilde{\rho }}_{\upsilon }{\;\tilde{v}}_{\infty }{\tilde{R}}_{P}/{\tilde{\mu }}_{\upsilon }<1$. In the marine environment, the hypothesis of laminar flow regime with Re<1 is suitable for slowly drifting, sinking or rising particles in deep waters, but also in the epipelagic zone of calm open ocean with low energy dissipation rate ($<10^{-5}cm^{2}/s^{3}$) [42]. However, an upper bound is set on the applicable particle size and velocity, ${\;\tilde{v}}_{\infty }<\left({\tilde{\mu }}_{\upsilon }/{\tilde{\rho }}_{\upsilon }\right){\tilde{R}}_{P}^{-1}$. Considering that the kinematic viscosity of seawater is about ${\tilde{\mu }}_{\upsilon }/{\tilde{\rho }}_{\upsilon }\sim 10^{-2}cm^{2}/s$ [37], the low Reynolds condition is satisfied by individual phytoplankton cells (Table 1 in [62]), as well as marine snow microparticles.

**Figure 8:**
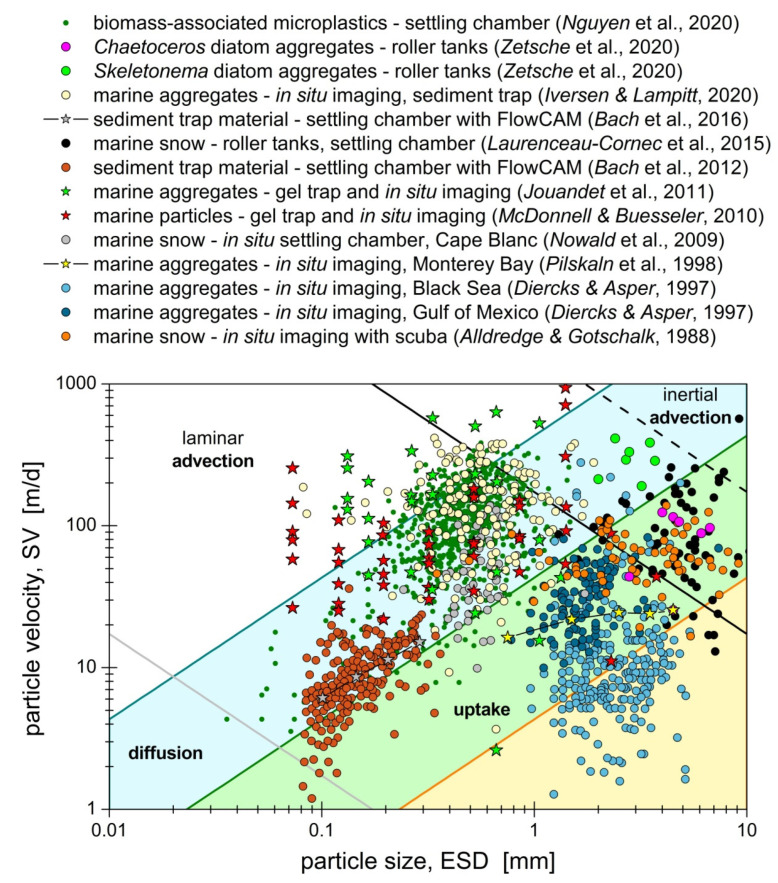
Phase diagram in the velocity–size space delineating the domain of prevalence for the fundamental transport processes that shape the nutrient field around sinking marine aggregates. The symbols represent experimental data for the sinking velocity (SV) and the equivalent sphere diameter (ESD) of individual aggregates (circles) or populations of aggregates (stars). The lines represent theoretical boundaries established by timescale conditions. The black lines correspond to Re = 1 (solid) and Re = 10 (dashed), with a kinematic viscosity of ${\tilde{\mu }}_{\upsilon }/{\tilde{\rho }}_{\upsilon }=10^{-2}cm^{2}/s$ for seawater. The solid grey line corresponds to Pe = 1 with ${\tilde{D}}_{A\upsilon }=10^{-5}cm^{2}/s$. The colored lines correspond to Pe/Da=100 with uptake timescale ${\tilde{\tau }}_{U}=10s$ for dark cyan, ${\tilde{\tau }}_{U}=100s$ for dark green, and ${\tilde{\tau }}_{U}=1000s$ for orange. The plume lifetime is in the range of >1000 *s* in the yellow-shaded area, 100–1000 *s* in the green-shaded area, 10–100 *s* in the cyan-shaded area, and <10 *s* in the non-shaded area. Microbial uptake and plume quenching are important for data falling in the green-yellow areas, modest in the cyan area, and negligible in the non-shaded area. Figure citations: [22,86,87,88,89,90,91,92,93,94,95,96,97].

**Figure 9:**
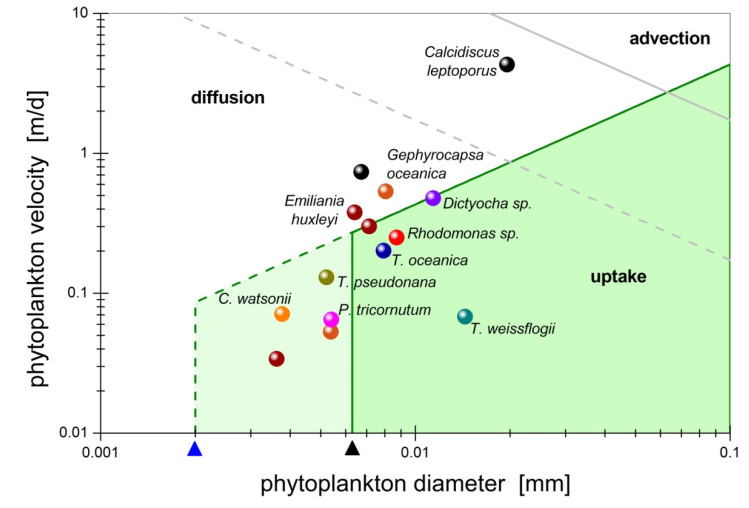
Phase diagram in the velocity-size space delineating the domain of prevalence for the fundamental transport processes that shape the nutrient field around individual phytoplankton cells. The symbols correspond to experimental data (Table 1 in [86]). The lines represent theoretical boundaries estimated by timescale conditions. The grey lines correspond to Pe = 1. The dark green lines correspond to Pe/Da = 100 with uptake timescale ${\tilde{\tau }}_{U}=100s$. The arrowheads point at critical diameters corresponding to $Da=10^{-4}$. The nutrient diffusivity is ${\tilde{D}}_{A\upsilon }=10^{-5}cm^{2}/s$ for small solutes (black arrow, solid lines) and ${\tilde{D}}_{A\upsilon }=10^{-6}cm^{2}/s$ for large solutes (blue arrow, dashed lines).

As shown in Figure 8, the above timescale conditions are satisfied by sinking marine aggregates with size (diameter) in the range of 0.1 mm to 10 mm and velocity less than 40 m per day. For larger particles or higher velocity, the nutrient field is dominated by advection. For smaller particles, like individual phytoplankton cells, molecular diffusion also becomes important (Figure 9). In agreement with Jackson’s [48] analysis, a large fraction of marine aggregates generates short-lived plumes with lifetime below 100 *s* (cyan-shaded and non-shaded areas in Figure 8). However, there also exists a significant fraction of marine aggregates with longer plume lifetime, ${\tilde{\tau }}_{plm}>100s$, for which uptake by microbes contributes significantly in shaping the nutrient field (green and yellow shaded areas in Figure 8). Consequently, plume quenching is impactful for slow-sinking large particles with small solutes (e.g., marine snow releasing hydrolyzed amino acids [25]), and for small particles with large solutes (e.g., phytoplankton cells excreting polysaccharides of high molecular weight [87]).

The morphology and composition of marine aggregates strongly affect their sinking behavior [89,96], and underpin the significant scatter of data observed in Figure 8. Marine aggregates consist of exopolymer substances (EPS), microorganisms, mineral particles and detritus [4,5,6,7,8]. The EPS matrix, inclusive of transparent exopolymer particles (TEP), acts as a glue and provides cohesion and structural stability to the aggregate. High EPS content is typically correlated with large, slow-sinking aggregates. For example, the sinking velocity of diatom aggregates [88,98], biomass-associated minerals [99] and microplastics [22] was found to decrease with increasing EPS content. This interesting trend is partly attributed to the interstitial water, which is physico-chemically absorbed by the EPS matrix during aggregate formation. Marine gels, the precursors of aggregates [5], usually form in low salinity waters of the epipelagic layer or freshwater rivers, hence binding interstitial water of low excess density. Thereafter, as the aggregates sink through denser seawater, they slow down and increase their residence time or even get trapped in strong pycnoclines [83,100,101]. Mineral particles also affect the mechanics and settling behavior of the aggregates [18,99], with higher mineral content resulting in stiffer aggregates of high excess density and sinking velocity. For example, aggregates affected by turbid estuarine waters of the Mississippi river were rich in clay and sank faster than similarly sized aggregates formed in offshore waters of the Black Sea [96]. Based on the above, plume formation and quenching is expected to be more pronounced for aggregates with high EPS and low mineral content.

The aggregate morphology is another key determinant of particle transport in the sea. A wide range of aggregate shapes has been observed in situ with underwater video cameras and microscopes [83,102]. In modeling studies, marine aggregates are typically represented by spherical particles [37,43,44,66,101], because nearly spherical aggregates exist in situ [97] and the sphere is the simplest shape that can serve as a reference for comparisons between different studies. What is more important, recent experimental work demonstrated that the rigid sphere model provides satisfactory agreement with the measured dissolution pattern around individual aggregates, either synthetic or natural (see, for instance, Figure 1d in [31], Figure 6 in [44], and Figure 9 in [88]). Furthermore, *McDonnell & Buesseler* [93] successfully described the observed variability for the velocity of sinking aggregates by combining the rigid sphere model for small particles of high sphericity with the rigid cylinder model for elongated particles of low sphericity. Based on the above information, the rigid sphere model is a reasonable first approximation towards capturing salient features of plume formation and quenching in marine waters. Subsequent modeling studies could investigate the process for marine aggregates with soft material behavior [103] and fractal geometry [22].

### 3.7. Further Perspective on Plume Quenching Implications

Ocean-level models of the biological carbon pump rely on the dichotomy between fast- and slow-sinking particles [1]. In contrast to rapidly sinking, slow-sinking particles have low density, high porosity and negligible mineral content. Over the entire spectrum of sinking rates, the particulate flux declines with depth [9]. Fast-sinking particles are susceptible to fragmentation [104], whereas slow-sinking ones are consumed by surface- attached microbes [11,31] and zooplankton [35]. Recent lines of evidence leverage the high abundance and strong control of slow-sinking particles over biogeochemical cycles [11,105,106,107,108]. For instance, in situ measurements revealed that slow-sinking particles (<30 m/d) prevail in the vertical POM flux in the Atlantic Ocean [107], and small particles (128–512 μm) control the rate of anaerobic ammonium oxidation in the hypoxic zone of the Southeast Pacific Ocean [108]. In this context, the microscale process of plume feeding and quenching by free-living bacteria underpins a large potential impact on the rates of particle degradation and nutrient depletion and, optimally, should be incorporated in ocean-level models of POM and DOM transport.

In this work, we investigated the base effects of plume quenching by considering uniformly distributed bacteria around the particle. This setup is reasonable because the oceanic microbiome is typically dominated by non-motile bacteria, like *Pelagibacters* of the SAR11 clade [76,109], for which there is no driving force to significantly disrupt a uniform spatial distribution [46,47]. The fraction of motile chemosensing bacteria, which are able to respond to nutrient gradients and cluster around particles [40,110], is usually low (<10%) [111]. However, chemotactic bacterial clustering is expected to be important in eutrophic coastal waters, after episodic terrestrial runoffs and algal blooms [42,43]. Under conditions of such particulate blooms, the effects of plume quenching may be further amplified and deserve to be examined separately. Future research should also address the impacts of finite Reynolds number and irregularly shaped particles.

## 4. Conclusions

Our computational analysis shows that microbial uptake can significantly quench the extent and intensity of the nutrient plume in the wake of slow-moving particles when the uptake timescale is shorter than the plume lifetime or, equivalently, when Pe/Da < 100. In the context of marine ecosystems, plume quenching is expected to be substantial for individual phytoplankton cells as well as marine snow aggregates with sizes in the range of 0.1–10 mm and velocities up to 40 m/d. Our single-particle analysis could be used to parameterize ocean-level models [15,16] and, ultimately, lead to improved predictions of POM transport, nutrient transformations and microbial growth dynamics.

## Figures and Tables

**Table 1:** Model parameters.

Symbol	Range	Units	Description
${\tilde{R}}_{P}$	0.05–5	mm	particle radius
${\tilde{v}}_{\infty }$	0–100	m/d	reference velocity ^(1)^
${\tilde{D}}_{A\upsilon }$	10^−5^–10^−7^	cm^2^/s	nutrient diffusivity ^(2)^
${\tilde{B}}_{\upsilon }^{\infty }$	10^4^–10^7^	cells/mL	bacterial abundance ^(3)^
${\tilde{\alpha }}_{S}$	0.01–10	pL/(cell⋅s)	nutrient affinity ^(4)^
Pe	0–1000	−	Péclet number
Da	0–10	−	Damköhler number ^(5)^

^1^ see Section 3.6 and references therein; ^2^ *Smriga* et al. [43]; ^3^ *Azam* et al. [61]; ^4^ see Section 3.3; and ^5^ the uptake coefficient is estimated as ${\tilde{k}}_{\infty }={\tilde{\alpha }}_{S}{\tilde{B}}_{\upsilon }^{\infty }$ [43,45,46,47,48].

**Table 2:** Literature review for experimental data delineating the range of the bacterial affinity for organic and inorganic nutrients. Symbols: ${\tilde{u}}_{max}$ is the maximum uptake rate per cell, ${\tilde{K}}_{S}$ is the half-saturation constant, ${\tilde{\alpha }}_{Sb}$ is the per-biomass affinity, and ${\tilde{\alpha }}_{S}$ is the per-cell affinity.

**Bacteria**	**Substrate**	${\tilde{u}}_{max}$	${\tilde{K}}_{S}$	${\tilde{\alpha }}_{Sb}$	${\tilde{\alpha }}_{S}$	Reference
		$\left[\frac{nmol}{(mg-cell)\cdot h}\right]$	[nM]	$\left[\frac{L}{(mg-cell)\cdot h}\right]$	$\left[\frac{pL}{cell\cdot s}\right]$	
*Vibrio splendidus* ^a^	phosphate	1330	100 ^d^	13.3	4.27	[67]
*Vibrio splendidus* ^b^	phosphate	455	100 ^d^	4.55	0.72	[68]
*Roseobacter algicola*	phosphate	364	100 ^d^	3.64	2.37	[68]
*Nesjøen lake mixed culture*	phosphate	310	96	3.23	0.79	[69]
*N. maritimus* SCM1	ammonium	5231	134	39.04	0.63	[70]
*Nitrospira inopinata*	ammonium	2596	840	3.09	0.11	[71]
*Escherichia coli* ML308 ^a^	glucose	2349	597	3.93	1.72	[49]
*Escherichia coli* ML308 ^b^	glucose	5390	13,000	0.41	0.27	[49]
*Spirillum* sp. DSM 1109	lactate	397	5800	0.07	0.010	[72]
*Cycloclasticus oligotrophus*	toluene	13,132	651	20.17	1.40	[73]
*Pseudomonas putida* mt2	m-xylene	3750	340	11.03	1.01	[74]
	p-xylene	4710	1300	3.62	0.33	[74]
	toluene	4230	7700	0.55	0.050	[74]
*Burkholderia* sp. PS14	TCB ^c^	667	2883	0.23	0.026	[75]
*Pelagibacter* HTCC1062	GBT ^c^	12.63	0.89	14.19	0.141	[76]
*Pelagibacter* HTCC7211	GBT ^c^	14.92	1.85	8.06	0.080	[76]
*Vibrio* sp. strain S14	leucine	35.70	760	0.05	0.033	[77]
*Marinobacter arcticus*	leucine	53.36	198	0.27	0.025	[78]

^a^ acclimated chemostat-grown cells, ^b^ non-acclimated batch-grown cells, ^c^ abbreviations for TCB = 1,2,4-trichlorobenzene, GBT = glycine betaine, ^d^ based on Vadstein et al. [69].

## Data Availability

All data needed to evaluate the conclusions in the paper are present in the paper. Additional data related to this paper may be requested from the authors.
